# Fatal pulmonary embolism related to anti-psychotics: forensic implications. About four autopsy cases with review of the literature

**DOI:** 10.1192/j.eurpsy.2024.1210

**Published:** 2024-08-27

**Authors:** M. Kacem, W. Bouali, Y. Mahjoub, S. Brahim, L. Zarrouk

**Affiliations:** Psychiatric department, Taher Sfar Hospital of Mahdia, Mahdia, Tunisia

## Abstract

**Introduction:**

The association between the intake of antipsychotic drugs and the occurrence of thromboembolic complications is widely described in the literature. The occurrence of this complication may call into question the medical responsibility of the attending physician.

**Objectives:**

The objective of this work is to describe the physiopathological mechanisms involved in the occurrence of thromboembolic complications in a patient under antipsychotic treatment, whether or not associated with physical restraint and to discuss the forensic implications.

**Methods:**

Our study is retrospective on cases of fatal pulmonary embolism, discovered at autopsy, in connection with the taking of antipsychotics. The autopsies were carried out in the Department of Forensic Medicine of the Tahar Sfar University Hospital in Mahdia. The cases were collected over a period of 04 years. A review of the literature was carried out. We only selected articles published until February 2021 and dealing with cases of patients on antipsychotics, diagnosed with pulmonary embolism by performing a chest CT scan or during an autopsy.

**Results:**

915 autopsy cases were performed during the study period. Twenty cases of pulmonary embolism, discovered at autopsy, were collected. Four cases were related to the taking of antipsychotics (incidence 0.004%), including two men and two women, aged between 25 and 52 years. They were all on antipsychotic treatment for at least 5 years, with the exception of one case who was put on 3 antipsychotics, 7 days before his death, with indication of physical restraint. After analysis of the memorial data, the external examination and the autopsy, the results of additional examinations, the death was attributed, in the 4 cases, to a massive fibrino-cruoric pulmonary embolism. A selection of 45 studies regarding thromboembolic complications associated with taking antipsychotics, was included in the final review.

**Conclusions:**

The reported cases provided additional evidence on the involvement of antipsychotics in the occurrence of thromboembolic complications. Psychiatrists should be careful when prescribing these treatments. The establishment of therapeutic guidelines, taking into account the thromboembolic risk factors, becomes essential, in order to avoid the occurrence of a complication which could engage both the vital prognosis of patients and the responsibility of the physician.

**Disclosure of Interest:**

None Declared

